# Supplementary material for: An O(n) method of calculating Kendall correlations of spike trains
Source: PLoS One. 2019 Feb 14;14(2):e0212190. doi: 10.1371/journal.pone.0212190 (PMC6375604; doi:10.1371/journal.pone.0212190)
Supplement: S1 Code — (PDF) [file pone.0212190.s001.pdf]

S1 Code:

A MATLAB function using the method presented here is available for download at https://github.com/william-redman/Kendall-Correlation-for-Large-Spike-Trains.
