# Supplementary material for: An O(n) method of calculating Kendall correlations of spike trains
Source: PLoS One. 2019 Feb 14;14(2):e0212190. doi: 10.1371/journal.pone.0212190 (PMC6375604; doi:10.1371/journal.pone.0212190)
Supplement: S1 Text — (PDF) [file pone.0212190.s002.pdf]

S1 Text: Implementation of Knight's method

Our implementation of Knight's method that was used to compare to our method is also available on Github (see S1 Code). Below, we outline the steps we used.

Knight's algorithm can be summarized in the following three steps [1]:

1. Sort X. Break any ties in X according the values in Y. Use the sorting index of X to rearrange Y (denoted Y').

2. Use mergesort on Y' and count the number of inversions necessary to sort Y'.

3. Use the number of inversions to calculate the Kendall correlation.

Because we were interested only in spike trains, we achieved the sorting of X (first step) without using mergesort or any related sorting algorithm. The sorted X was simply a vector with n_{x_0} zeros followed by n_{x_1} ones, where n_{x_0} (n_{x_1}) is the number of zeros (ones) in X. Similarly, Y' is a vector with $n_{x_0 y_0}$ zeros followed by $n_{x_0 y_1}$ ones followed by $n_{x_1 y_0}$ zeros followed by $n_{x_1 y_1}$ ones, where $n_{x_0 y_0}$ is the number of places where Y had zeros and X had zeros, etc. This follows from the fact that all ties in X were settled by Y. Note that this approach to sorting X and Y is linear in n because it requires only a few passes through X and Y to find $n_{x_0}, n_{x_1}, ...$

We used a basic, third party implementation of mergesort [2] that was slightly modified to correctly account for the inversion count, α.

Because we were working with spike trains, α is equivalent to K^- in our method. K^+ can be shown to equal $n_{x_0 y_0} \cdot n_{x_1 y_1}$. Because we had already found $n_{x_0 y_0}$ and $n_{x_1 y_1}$ to get Y', calculating K^+ required no new operations on Y'. Therefore, the Kendall correlation using Knight's method was

$$\tau = \frac{(n_{x_0 y_0} \cdot n_{x_1 y_1}) - \alpha}{\sqrt{n_0 - n_1}\sqrt{n_0 - n_2}} \tag{1}$$

where n_0, n_1, and n_2 were defined earlier in the paper.

References

[1] Knight WR. A computer method for calculating Kendall's tau with ungrouped data. J. Am. Stat. Assoc. 1966; 61 (314): 436–439.

[2] https://stackoverflow.com/questions/31557266/counting-inversions-in-matlab-using-mergesort
